# Supplementary material for: Comparative Chloroplast Genomes and Phylogenetic Relationships of True Mangrove Species Brownlowia tersa and Brownlowia argentata (Malvaceae)
Source: Curr Issues Mol Biol. 2025 Jan 23;47(2):74. doi: 10.3390/cimb47020074 (PMC11854185; doi:10.3390/cimb47020074)
Supplement: Supplementary file 1 [file cimb-47-00074-s001.zip › Figure S1.pdf]

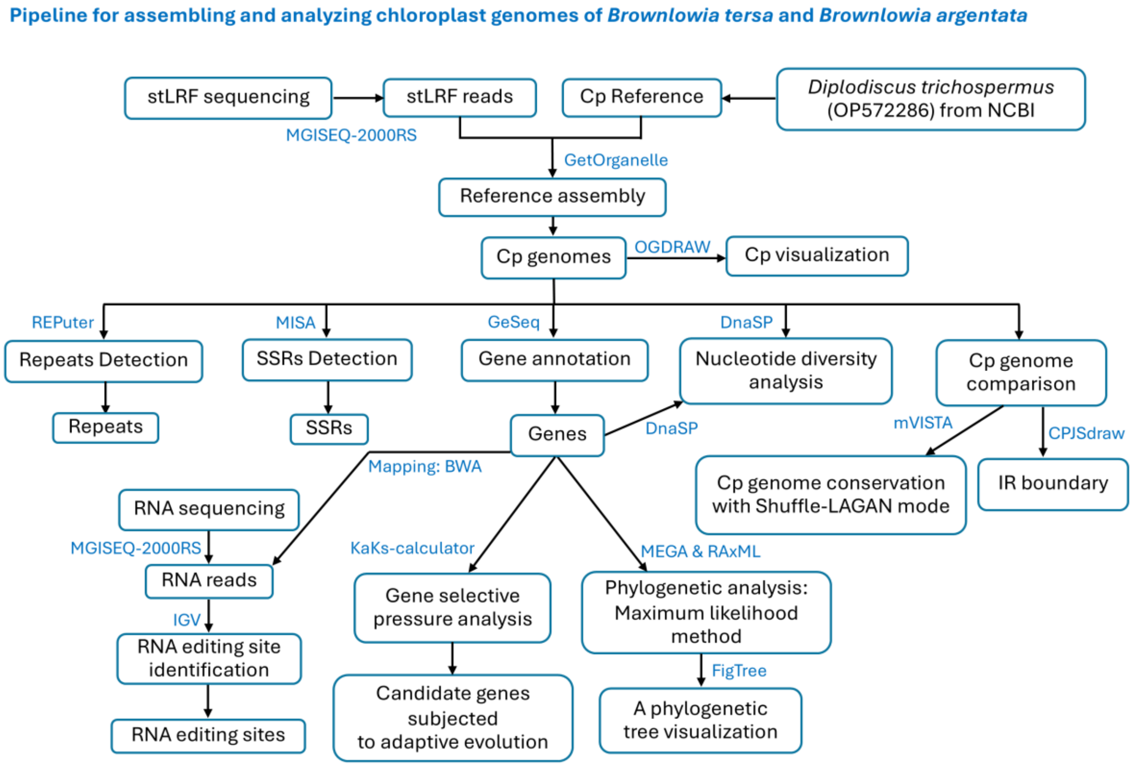

Figure S1. A flow chart for assembling and analyzing chloroplast genomes of *Brownlowia tersa* and *Brownlowia argentata.*
